# Multiphase Partitioning of Estrogens in a River Impacted by Feedlot Wastewater Discharge

**DOI:** 10.3390/toxics12090671

**Published:** 2024-09-14

**Authors:** Kuo-Hui Yang, Hao-Shen Hung, Wei-Hsiang Huang, Chi-Ying Hsieh, Ting-Chien Chen

**Affiliations:** 1 Department of Environmental Science and Engineering, National Pingtung University of Science and Technology, Pingtung 91201, Taiwan; pepea@mail.npust.edu.tw (K.-H.Y.); newhon16@yahoo.com.tw (H.-S.H.); carl7510@mail.npust.edu.tw (W.-H.H.); 2 Disaster Prevention and Mitigation Technology Research Center, General Research Service Center, National Pingtung University of Science and Technology, Pingtung 91201, Taiwan

**Keywords:** multiphase partitioning, estrogens, suspended particulate matter (SPM), colloid, soluble

## Abstract

Estrogens in river systems can significantly impact aquatic ecosystems. This study aimed to investigate the multiphase partitioning of estrogens in Wulo Creek, Taiwan, which receives animal feedlot wastewater, to understand their distribution and potential environmental implications. Water samples were separated into suspended particulate matter (SPM), colloidal, and soluble phases using centrifugation and cross-flow ultrafiltration. Concentrations of estrone (E1), 17β-estradiol (E2), and estriol (E3) in each phase were analyzed using LC/MS/MS. Partition coefficients were calculated to assess estrogen distribution among phases. Estrogens were predominantly found in the soluble phase (85.8–87.3%). The risk assessment of estrogen equivalent (EEQ) values suggests that estrogen concentration in water poses a higher risk compared to SPM, with a majority of the samples indicating a high risk to aquatic organisms. The colloidal phase contained 12.7–14.2% of estrogens. The log *K_COC_* values (4.72–4.77 L/kg-C) were significantly higher than the log *K_OC_* and log *K_POC_* values (2.02–3.40 L/kg-C) for all estrogens. Colloids play a critical role in estrogen distribution in river systems, potentially influencing their fate, transport, and biotoxicity. This finding highlights the importance of considering colloidal interactions in assessing estrogen behavior in aquatic environments.

## 1. Introduction

With the rapid development of industrialization and urbanization, the presence and distribution of endocrine-disrupting substances in aquatic environments have become a global environmental issue [1,2,3,4]. Studies have shown that natural estrogens, such as estrone (E1), 17β-estradiol (E2), and estriol (E3), even at extremely low concentrations (in the ng/L range) [1,5,6,7], can have serious effects on the reproductive systems of aquatic organisms, such as gender ratio imbalance and reduced reproductive capacity [5,6,8,9].

The main sources of natural estrogens are human and animal excreta [1,2,5,10,11], sewage discharges from livestock facilities, and agricultural fertilization, which are the primary pathways for estrogen entry into water bodies [12,13,14,15,16,17]. The distribution of estrogens in water bodies poses a potential threat to ecological safety. Animal excreta may account for 50% to 90% of natural estrogens in the environment [1,18,19,20,21].

The distribution of natural estrogens in aquatic environments is not limited to the dissolved phase; they also interact with suspended particulate matter (SPM), colloids, and sediments [6,22,23,24,25,26]. The distribution between these different phases affects the migration, transformation, and fate of estrogens, further impacting the quality of benthic organisms and sediments [22,27,28,29,30]. For example, colloidal particles have a strong adsorption capacity for estrogens due to their high specific surface area and abundant organic carbon content, affecting their bioavailability and toxicity [6,22,27,30,31]. Colloids can transport estrogens over long distances, thus affecting the quality of water bodies far from pollution sources [29,32]. Additionally, estrogens in the dissolved phase are more readily absorbed by aquatic organisms, increasing their toxicity risk [6,31].

Although studies have investigated the concentration and distribution of estrogens in water bodies, most have focused on the dissolved and particulate phases such as sediment and SPM, with relatively fewer studies on the colloid phase [6,28,30,33]. Furthermore, colloids of different origins and properties exhibit significant differences in their adsorption capacity for estrogens, making a comprehensive assessment of the multiphase distribution of estrogens in aquatic environments more complex [6,23,28,29,34].

Studies indicate that colloids are pivotal in the distribution of estrogens in aquatic environments. According to Huang et al. [31], the proportion of estrogens (E1, E2, and E3) in SPM fluctuates between 0 and 19.8%, 0 and 18.9%, and 0 and 6.9%, respectively. Conversely, in the colloid phase, estrogen percentages range from 14.6% to 35.5% for E1, 7.1% to 39.5% for E2, and 19.3% to 46.4% for E3. These findings underscore a notable presence of estrogens in colloids compared to SPM, illustrating the crucial role of colloids in the aquatic transport and behavior of estrogens.

Additionally, research reveals significant variations in the binding or partitioning coefficients of estrogens between distinct phases, namely SPM and the liquid phase (*K_OC_*), SPM and the dissolved phase (*K_POC_*), and colloid and the dissolved phase (*K_COC_*). Huang et al. [31] documented average log *K_OC_*, log *K_POC_*, and log *K_COC_* values for E1, E2, and E3, demonstrating the differential affinities estrogens exhibit towards diverse phases. Similarly, Nie et al. [29] provided comparative log *K_OC_*, log *K_POC_*, and log *K_COC_* values for these estrogens, highlighting distinct partitioning behaviors in aquatic environments.

Crucially, *K_COC_* values were found to be one to three orders of magnitude higher than *K_OC_* and *K_POC_*, suggesting that colloids possess a considerably higher affinity for estrogens compared to suspended particulate matter. These partition coefficients’ variability is significantly influenced by the source of emissions and the physicochemical characteristics of both colloids and SPM in aquatic systems. These insights point to the necessity of incorporating colloids into assessments of estrogen distribution and environmental behavior in water bodies.

Despite existing research indicating the multiphase distribution characteristics of natural estrogens in aquatic environments [29,31,34], there are still challenges and shortcomings in current research. The multiphase distribution characteristics of estrogens under different environmental conditions are not fully understood [6,29]. A thorough investigation of the multiphase distribution of natural estrogens in aquatic environments will help reveal their environmental behavior and fate, understand the distribution and transformation patterns of estrogens between different phases, and provide a scientific basis for pollution control and remediation [6,28,34].

This study seeks to examine the distribution of natural estrogens in a river that receives significant wastewater discharges from animal husbandry. The specific objectives are as follows: 1. Quantify the concentrations of estrone (E1), 17β-estradiol (E2), and estriol (E3) in SPM, filtrate, colloidal, and soluble phases. 2. Determine the partition coefficients (*K_OC_*, *K_POC_*, *K_COC_*) of these estrogens among different phases. 3. Assess the risk of estrogen in water and SPM. This comprehensive analysis of estrogen distribution will contribute to a better understanding of its behavior in aquatic environments.

## 2. Materials and Methods

### 2.1. Chemical Reagents

Estrone (E1, 99%), 17β-estradiol (E2, 99%), and estriol (E3, 97%) were procured from Cerilliant (Oakville, ON, Canada) Methanol of HPLC grade (99%) was sourced from Tedia Company Inc., (Fairfield, OH, USA), Ammonium hydroxide of ACS grade (28–30%) was obtained from J.T. Baker (Phillipsburg, NJ, USA). Additionally, acetonitrile of HPLC grade (99.9%) was acquired from (Charlotte, NC, USA). Sulfuric acid of EP grade (95%) was purchased from Union Chemical Works, Taiwan.

### 2.2. Sampling Site

The water and SPM samples were taken from two specific sites along Wulo Creek, which is a tributary of the Gaoping River in Pingtung County, Taiwan. The Wulo Creek Basin experiences heavy rainfall during the wet season, with an average annual precipitation of around 2500–3000 mm. The temperature in the area ranges from 15 °C to 35 °C throughout the year, with the highest temperatures usually recorded in July and August. The high flow occurs during the wet season, from May to September, while the low flow occurs during the dry season, from October to April. As a result, this study’s sampling period is from January to April each year to avoid rainy days.

This particular area is known for its large-scale animal husbandry practices, supporting a significant population of around 4500 cattle, 500,000 pigs, 3 million laying hens, and 5 million broilers.

**Figure 1:**
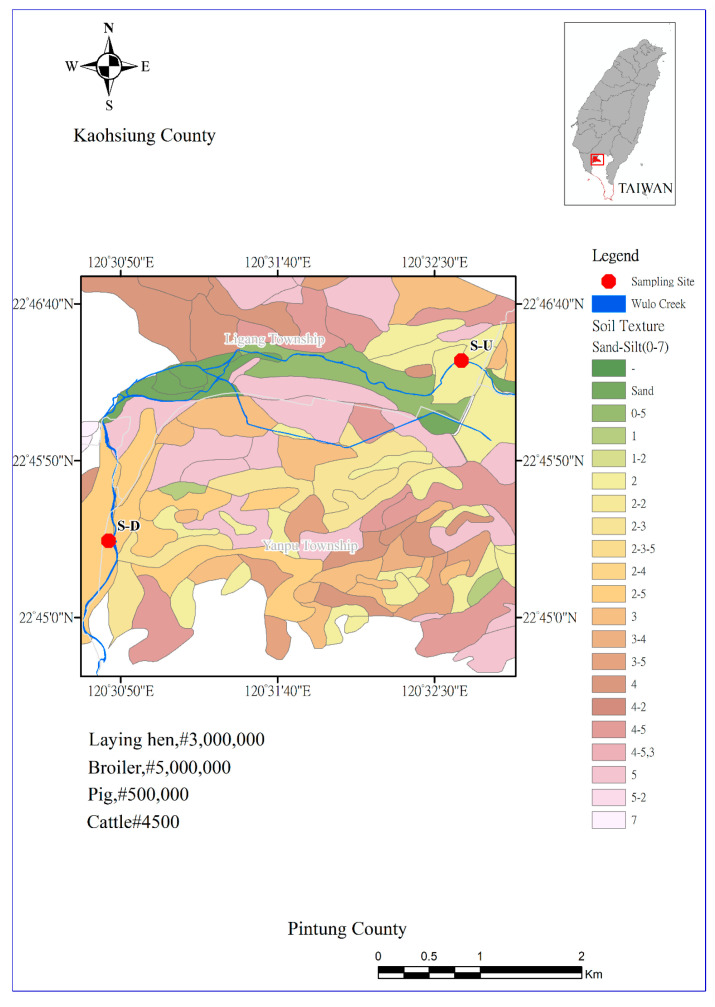
Geographical map of sampling site locations (adapted from Hung et al. [37], and Ministry of Agriculture, Republic of China (Taiwan)).

The selection of these sampling sites was based on previous research by Chen et al. [35] and Hung et al. [36], which identified these locations (WL-1 and WL-4) as areas with high estrogen concentrations. These two sites are about 5 km apart. Figure 1 shows the geological map and sampling sites, and # indicates the number of animals. 

In our study, the upstream site (referred to as site-S-U) is located near the main discharge points of animal wastewater, while the downstream site (referred to as site-S-D) is further downstream. Both sampling sites can be accessed via bridges across the creek. The samples were collected from the central location of the creek to ensure a representative sampling of the water and SPM. This strategic approach was used to capture the overall environmental conditions and pollutant levels present in the water body. A total of 25 validated samples were collected for water and SPM. The detailed sampling description is provided in the Supplementary Information. The collected samples underwent basic water chemistry measurements, the results of which are listed in Table S1. The water quality, as per the River Pollution Index (RPI), was classified as “Moderately Polluted”, reflecting the properties of animal wastewater.

### 2.3. Sampling Procedure and Treatment

The sampling of water and SPM involved using a peristaltic pump (Subaru, EY 20-3D-5.0 HP, Shibuya-ku, Tokyo, Japan) to extract river water onto the bridge and into a clean, pre-washed 20 L sampling bucket. Approximately 50–75 buckets, totaling around 1000–1500 L of water, were collected each time. To prevent estrogen degradation, 10 g of sodium azide was added to each full bucket of water. The suspended solid was separated from water samples using a continuous high-speed centrifuge (20,000 rpm) (Quantai Iron Works, LOS100LS, Taiwan). The water sample underwent solid–liquid separation by using a peristaltic pump (Cole-parmer MasterFlex, 7553-70, Gelsenkirchen, Germany) to feed the original water sample into the centrifuge tube of the continuous separation machine (Quantai Iron Works, LOS100LS, Taiwan), maintaining a flow rate of approximately 0.5 L/min. The high-speed centrifuge causes the water sample to adhere to the centrifuge tube wall, effectively collecting suspended solids; for each sample collection, the separation process took over 1 day each time. After solid–liquid centrifugal separation, the collected suspended solids were scraped off the centrifuge tube wall and placed into brown wide-mouth bottles, removing non-SPM samples such as small stones. These SPM samples were then placed in a freezer, and after 24 h, approximately 50 g of frozen SPM samples were further processed using a freeze dryer (FDU-1200, EYELA, Tokyo, Japan). The water-containing SPM sample was drained at −50 °C, under 5 Pa vacuum, and the freeze-drying time was about 3–5 days (depending on the solids’ moisture content). The freeze-dried SPM samples were crushed and passed through a #20 mesh sieve (0.814 mm) to obtain the SPM sample.

After separating the solid and liquid components, the liquid samples were first filtered through a 0.7 μm coarse filter and then through a 0.22 μm fine filter using cellulose acetate membrane materials to remove suspended solids. Following the filtration, 12,000 mL of the filtered liquid was stored in a refrigerator at 4 °C. This was carried out in preparation for subsequent separation of the colloidal and dissolved phases and then solid-phase extraction (SPE).

### 2.4. The Separation Process of Water Samples

The liquid samples were filtered and then separated into colloidal and dissolved samples using a feed flow rate of 1.7–2.0 L/min. Throughout the separation process, the concentration volume factor was maintained at 10, and the volume ratio of dissolved sample (*V_dissolved_*) to colloidal sample (*V_colloid_*) was kept at 1:9. The mass recovery ratios (MB, %) for estrogens and DOC were calculated using Equation (1), while the mass percentages (MF_i_ %) of the colloidal and dissolved phases for estrogens and DOC were calculated using Equation (2) [38].

(1) $$\mathrm{MB}\;\left(\%\right)=\frac{\sum \;\left(\left(C_{dissolved}\times V_{dissolved}\right)+\left(C_{colloid}\times V_{colloid}\right)\right)}{C_{filtrate}\times V_{filtrate}}\times 100\;$$

(2) $${\mathrm{MF}}_{i}\left(\%\right)=\frac{C_{i}\times V_{i}}{\sum \;\left(\left(C_{dissolved}\times V_{dissolved}\right)+\left(C_{colloid}\times V_{colloid}\right)\right)}\times 100\;$$

where *C_i_* and *V_i_* represent the concentration and volume of dissolved organic carbon (DOC) and estrogens in both colloidal and dissolved samples. *C_filtrate_* and *V_filtrate_* denote the concentration and volume of DOC and estrogens in the filtrate solution. *C_colloid_* and *V_colloid_* refer to the concentration and volume of DOC and estrogens in the colloidal solution. Similarly, *C_dissolved_* and *V_dissolved_* indicate the concentration and volume of DOC and estrogens in the dissolved solution.

During the separation process, the ultrafiltration membrane must be carefully cleaned. This process involved using reverse osmosis water, neutral detergent, 0.5 N sodium hydroxide solution, and deionized water (DDW) in a specific sequence.

### 2.5. Pretreatment of Liquid and SPM Samples for Estrogen Analysis

A volume of 1000 mL of the filtered liquid was subjected to solid-phase extraction (SPE). First, the filtered liquid samples were acidified using sulfuric acid to achieve a pH of 3.0. The target compound was then concentrated by passing through SPE cartridges using Oasis HLB (200 mg/6 mL, 30 μm particle size, Waters, Milford, Massachusetts, USA), which were pre-conditioned with 6 mL of methanol and 6 mL of deionized water. The acidified liquid sample was passed through the SPE column at a flow rate of 3–6 mL/min. Subsequently, 5 mL of 5% methanol was used to elute the cartridges, followed by drying under vacuum for 30 min. The column was then eluted with 3 mL of 5% methanol/double-distilled water (DDW) and further with 6 mL of methanol. Finally, the cartridge was eluted with 6 mL of 2% acetonitrile/methanol (ACN/MeOH) containing 0.5% NH_4_OH.

The eluted sample was then evaporated using slowly blown nitrogen until it reached a near-dry state and subsequently redissolved with a 2 mL solution of ACN/DDW (*v*/*v* = 10/90). Finally, it underwent filtration through a 0.22 μm polytetrafluoroethylene syringe filter for LC/MS/MS analysis. It is important to note that this method was modified from previous studies to optimize the parameters [36,38,39,40].

To analyze the estrogen content of SPM, a modified method was used based on previous studies [41,42,43]. Initially, 2 g of freeze-dried SPM (#20 mesh screening) was mixed with a 10 mL solution of acetonitrile and distilled deionized water (ACN/DDI = 9:1). The mixture was then sonicated using a sonicator at 180–240 W and a frequency of 20 kHz for 5 s, followed by a 5 s pause, and then resumed for 12 min. The sample was subjected to sonic extraction twice. Afterward, the solution was centrifuged at 2000 rpm for 20 min to obtain the supernatant, resulting in a total extract of 20 mL. The extract was then concentrated to 2 mL by purging with nitrogen. This concentrated solution was then dissolved in 98 mL of distilled deionized water (DDW). Finally, the solution was processed and analyzed following the pretreatment steps outlined for estrogen-containing liquid samples.

### 2.6. LC/MS/MS Analysis Conditions

The analytes were separated using an Agilent 1200 HPLC system (Agilent Technologies, Palo Alto, CA, USA) with a Phenomenex Gemini C-18 Column (100  ×  2.0 mm, dp  =  3 μm) at a constant temperature of 40 °C. Injections of 25 μL were analyzed in a mobile phase consisting of ultrapure water with 0.05% ammonium hydroxide (A) and acetonitrile with 0.05% ammonium hydroxide (B). The chromatographic gradient started at a flow rate of 0.2 mL/min, maintaining 20% of component B for 1 min, increasing to 35% within 1.5 min, reaching 58% over another 1.5 min, and then held for 0.3 min. At 6.7 min, component B was increased to 85%, and the total analysis duration was 11 min. Retention time (Rt), parent ion production, fragmentation voltage, and collision voltage adhered to the parameters optimized for standard products as per Hung et al. [36], using an Agilent 6410 LC/MS/MS system. Negative-ion electrospray ionization (ESI) was employed. The procedure utilized dynamic multiple reaction monitoring (MRM) with a drying gas temperature of 325 °C, drying gas flow of 8 L/min, nebulizer pressure of 30 psi, and capillary voltage of 4000 V.

### 2.7. Quality Assurance and Control

The calibration curves for estrogen-containing samples spiked with varying concentrations of three target estrogens (0.5 to 200 µg/L) demonstrated strong linearity (R^2^ > 0.995). To ensure the absence of contamination and proper instrument performance, procedural blanks and sample replicates were analyzed for each batch. Recovery rates were determined using a standard addition method with 10 ng/L spiking concentration and ranged from 79% to 91%. The method’s detection limits were 0.3, 0.5, and 0.4 ng/L for E1, E2, and E3, respectively, in water (Table S2). The internal quality assay was maintained below 5%, while the intermediate assay was below 8% at the quantitation limit, meeting the acceptable criteria for environmental analysis. Detailed quality assurance data for each compound in DI water and the liquid samples can be found in Hung et al. [36]

### 2.8. Partition Coefficient Calculation

In this study, we evaluated three types of partition coefficients: K_OC_, K_POC_, and K_COC_. The calculation of K_OC_, K_POC_, and K_COC_ is performed according to Equations (3)–(5), respectively [26,30,31].

(3) $$K_{OC}=C_{SPM}/C_{filtrate}/f_{OC}\;$$

(4) $$K_{POC}=C_{SPM}/C_{dissolved}/f_{OC}\;$$

(5) $$K_{COC}=C_{colloid}/C_{dissolved}/DOC_{colloid}\;$$

where *C_SPM_*, *C_filtrate_*, *C_colloid_*, and *C_dissolved_* indicate estrogen concentrations of SPM and the estrogen concentrations of filtrate, colloid, and dissolved solutions. *f_oc_* and *DOC_colloid_* indicate the TOC fraction of SPM and the DOC concentration of colloid.

### 2.9. 17β-Estradiol Equivalent (EEQ) Calculation

To assess the potential risks of estrogenic activity to aquatic organisms, estradiol equivalents (EEQs) in the filtrate and SPM collected from Wulo Creek were calculated.

The EEQ in filtrate water of the analyzed estrogens was calculated following Equation (6):

(6) $$\;EEQ=\sum EEQ_{i}=\sum \;\left(C_{i}\times EEF_{i}\right)\;$$

The risk assessment for the SPM was carried out by converting the estrogenic activities of estrogens into their corresponding EEQs in water, which were expressed as the bioavailable fractions of the estrogens in the SPM. The *EEQ_water_* in the SPM was calculated following Equation (7) [44,45]:

(7) $$EEQ_{water}\;\left(\mathrm{ng}/L\right)=\sum EEQ_{i}=\sum \;\left(1000\times C_{s,i}\;\left(ng/g\right)\times EEF_{i}\right)/K_{oc,i}\;\left(L/kg\right)$$

where *EEQ_i_* is the EEQ value of the selected estrogen i, and *C_i_* and *C_*s*,*i*_* are the concentration of the selected estrogen i in filtrate and SPM. *EEF_i_* is the estrogenic equivalent factor relative to E2 and the EEF values of E1, E2, and E3 were 0.25, 1, and 5.9 × 10^−3^, respectively [46]. *K_oc,i_* is the organic carbon standardized partitioning coefficient of chemical i between SPM and filtrate.

### 2.10. Statistical Analysis

In this study, various tests were conducted using S-Plus software (version 6.2) at a significance level of *p* < 0.05. The *t*-test method was used to compare estrogen differences between two groups, and the ANOVA test method was used for comparing differences between three groups, followed by Tukey’s post hoc test.

## 3. Results

### 3.1. Dissolved Organic Carbon Concentrations in Liquid Samples

In this study, 25 water samples were collected from sampling sites. Thirteen and twelve samples were taken from site-U and site-D, respectively. Table 1 lists the concentrations of dissolved organic carbon (DOC) and estrogens in the liquid samples. The samples include the filtrate (<0.45 μm), colloidal (1 kDa-0.45 μm), and soluble (<1 kDa) phases. The average DOC concentration in the total filtrate was 17.1 ± 9.7 mg/L at site-U, which was higher than the concentration of 8.2 ± 6.8 mg/L at site-D (*p* = 0.014). Site-U is located near animal feedlot wastewater discharge sources. This indicates that the river carries wastewater from agricultural areas with high concentrations of DOM, resulting in a high DOC concentration and poor water quality. Similar findings have been reported in other studies [35]. The lower DOC concentration at site-D was due to the aggregation and deposition of large particulate dissolved organic matter (DOM) as the river water flowed downstream.

The mass recovery ratios of filtrate separation into colloidal and soluble phase for organic carbon (OC) and estrogens were calculated using Equation (1). The OC recovery ratios ranged from 73% to 157%, with an average of 97% across all samples. Most of the OC recovery ratios fell between 80% and 120% for 20 out of 25 samples. These average OC recovery ratios were considered acceptable. For instance, in a study by Nie et al. [29] on water from the Huangpu River in China, the OC recovery ratios ranged from 88% to 128%, with an average of 108%. Similarly, Chuang et al. [38] found an average OC recovery ratio of 105% when separating river water into five types of size-fractionated DOM.

**Figure 2:**
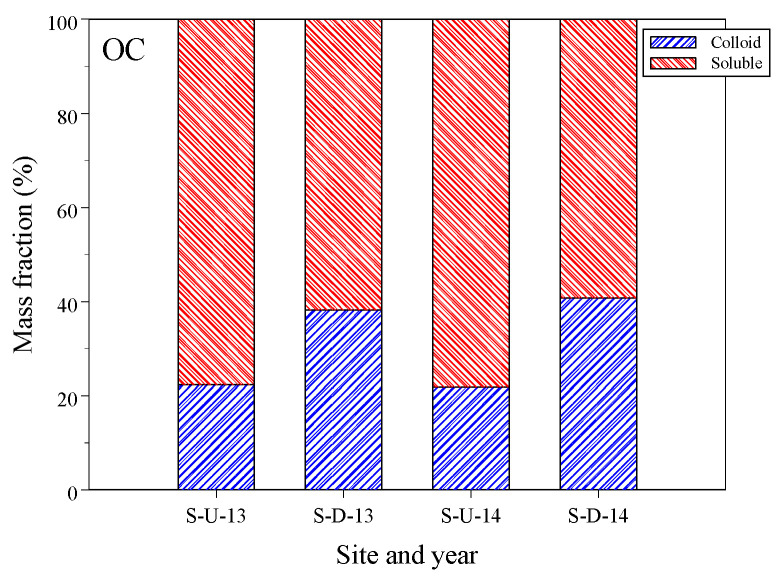
The mass percentages of organic carbon (OC) distributed on colloidal and soluble phases.

Figure 2 displays the OC mass percentages of colloidal and soluble phases using Equation (2) for two years and two sites. Despite the colloid having a higher DOC concentration compared to the soluble phase (Table 1), the volume fractions were 0.1 and 0.9 for colloidal and soluble phases, respectively. Consequently, the colloidal mass percentages were lower than those of the soluble phase. On average, the colloidal OC percentage was 31.5% with a standard deviation of 14.8%. At site-D, the colloidal percentage was significantly higher at 39.4% compared to site-U, which had a percentage of 22.0% (*p* = 0.003). The colloidal OC mass percentages in our study were slightly lower than the 32–55% range reported in freshwater DOM studies [38,47,48]. However, they are comparable to the findings of Chen et al. [30], whose study showed an average colloidal OC mass percentage of 30.0% with a standard deviation of 11.5%.

In our study, the low colloidal OC percentages indicate a low level of humification in the dissolved organic matter, suggesting a lack of synthesis of humic and fulvic acid- like organic matter due to the low humification extent of the water receiving animal wastewater discharge DOM. Additionally, the soluble phase of dissolved organic matter may aggregate to form a larger colloidal phase from site-U to site-D, increasing the percentage of the colloidal phase at site-D.

### 3.2. Estrogen Concentrations in Water Samples

The concentrations of three natural estrogens, E1, E2, and E3, were detected in all liquid phases. Table 1 lists the average estrogen concentrations of the filtrate samples. The order of total average concentrations of the filtrate was 153.0 ± 153.7 ng/L (E1) > 5.4 ± 3.7 ng/L (E2) > 4.2 ± 2.1 ng/L (E3), and the concentrations showed considerable variation. The sampling sites affected by the wastewater of animal feedlot discharge resulted in high estrogen concentrations [5,13,29,35,49,50]. The order and concentrations of estrogen were similar to animal-polluted river water [35,49], but the concentrations were higher than river water receiving effluent of STP wastewater [1,29,31]. The filtrate’s total average E1 concentration of 121.0 ± 112.0 ng/L at site-U was lower than the concentration of 187.6 ± 112.0 ng/L at site-D without significant difference (*p* = 0.302). Additionally, the E2 and E3 concentrations were insignificantly different at the two sites. The average E2 concentrations were 5.5 ± 4.0 ng/L and 5.3 ± 3.4 ng/L at site-U and site-D, respectively (*p* = 0.93). The average E3 concentrations were 4.0 ± 1.4 ng/L and 4.3 ± 2.7 ng/L at site-U and site-D, respectively (*p* = 0.68). According to Liu et al. [49] and Chen et al. [35], high estrogen concentrations were found upstream of Wulo Creek, with an E1 concentration exceeding 1000 ng/L, suggesting that animal wastewater could carry high estrogen concentrations to receiving water.

Table 1 also presents the estrogen concentrations in colloidal and soluble phases. The estrogen concentrations in these phases were measured directly and did not account for volume factors, leading to higher concentrations in the colloidal phase compared to the filtrate and soluble phases. After separation, the estrogen concentration of individual species in the colloidal and soluble phases was comparable to that in the filtrate.

The average estrogen mass recovery ratios were 88.8%, 92.0%, and 103.8% for E1, E2, and E3, respectively, in the filtrate separated into colloidal and soluble phases, which were within an acceptable range [27,29,30]. For example, in the partition study of estrogens between colloidal and soluble phases, the average mass recovery ratios were 131%, 99%, and 144% for E1, E2, and E3, respectively, during the separation processes [29]. Yan et al. [27] reported mass recovery ratios ranging from 64.2% to 117.8% for 27 emerging organic compounds in a separation test.

**Figure 3:**
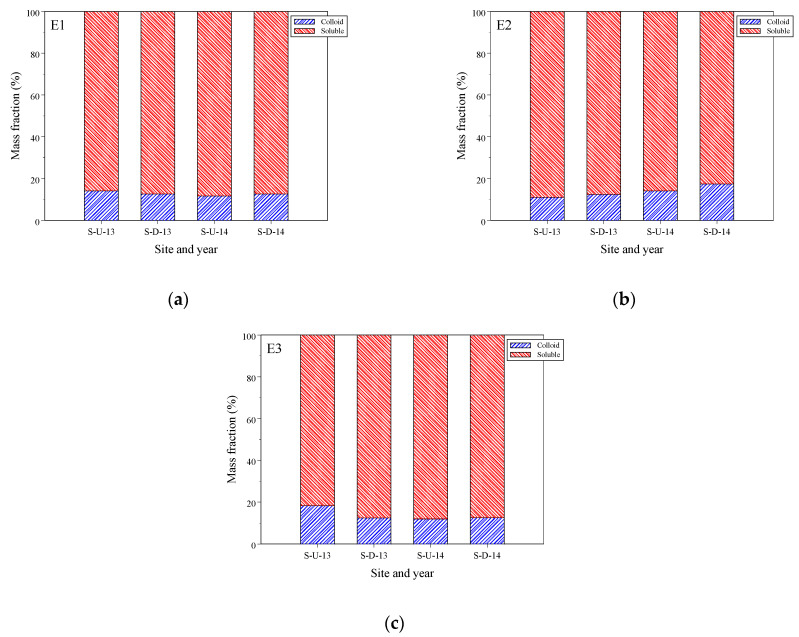
The mass percentages of estrogens distributed on colloidal and soluble phases: (**a**) (E1), (**b**) (E2), and (**c**) (E3).

Figure 3a–c illustrate the estrogen mass percentages of E1, E2, and E3, respectively, at site-U and site-D during the years 2013 and 2014. The overall average mass fractions of the colloidal phase were 12.7 ± 2.6%, 14.2 ± 5.5%, and 13.5 ± 5.5% for E1, E2, and E3, respectively. Additionally, the colloidal mass percentages of the estrogens did not show significant differences (*p* = 0.46); the mass percentage for each estrogen was not significantly different between 2013 and 2014 (*p* = 0.07–0.35) and between the two sampling sites (*p* = 0.32–0.97).

Previous studies on the multiphase partitioning of estrogens in in situ river water have demonstrated a wide range of percentages for colloid-bound estrogens. Nie et al. [29] reported ranges of 2.0–58.4%, 8.4–72.0%, and 3.4–62.7% for E1, E2, and E3, respectively. These percentages significantly differed from the present study due to variations in colloid sources. Huang et al. [31] found average colloid-bound phases of 26.3%, 15.1%, and 19.5% for E1, E2, and E3, respectively, in the Shaying River, China; these percentages were slightly higher than those observed in the present study. In a constructed wetland, Chen et al. [30] reported colloid-bound estrogen percentages ranging from 7.3% to 8.5% for E1, E2, and E3, which were lower than the percentages found in the present study. In four advanced wastewater treatment processes, Huang et al. [28] reported the mass percentages of three natural estrogens in different phases (suspended particulate matter, colloidal, and soluble phases); the colloid-bound fractions were 3.9–19.4%, 15.1–31.7%, and 54.0–77.8% for E1, E2, and E3, respectively. The percentages for E1 and E2 were similar to those in the present study, but the percentage for E3 was higher. The distribution of estrogen in the colloidal phase is influenced by sources, biochemical processes, and other factors [23,27,29,30,31,51,52].

### 3.3. Estrogen Concentrations in SPM

In this study, a total of 23 suspended particulate matter (SPM) samples were analyzed for estrogen concentrations. Eleven samples were taken from site-U and twelve from site-D. Table 2 presents the average SPM concentrations and estrogen concentrations extracted from SPM across the two sampling years and both sites. The overall average SPM concentration at site-U was 126.8 ± 33.8 mg/L, significantly higher than the 37.4 ± 19.2 mg/L observed at site-D (*p* < 0.001). As river water flows downstream from site-U to site-D, larger suspended particulates aggregate and settle into the sediment, thereby reducing the SPM concentration downstream.

Among the 23 SPM samples analyzed for estrogen concentrations, E2 was detected in 2013 but not in 2014. Some SPM samples had estrogen concentrations below the detection limit, with the number of detectable samples being 17 for E1, 6 for E2, and 13 for E3. Estrogen concentrations below the detection limit were considered zero in statistical calculations. The total average estrogen concentrations in SPM were 1.37 ± 1.19, 0.35 ± 0.73, and 1.19 ± 1.36 μg/kg for E1, E2, and E3, respectively, with E1 concentrations being significantly higher than E2 (*p* = 0.004). The E1 concentrations were 0.82 ± 1.10 μg/kg at site-U and 1.96 ± 1.01 μg/kg at site-D, while the E3 concentrations were 0.66 ± 1.12 μg/kg at site-U and 1.77 ± 1.40 μg/kg at site-D. Both E1 and E3 concentrations were higher at site-D than at site-U (*p* = 0.013 for E1; *p* = 0.042 for E3). This higher estrogen concentration at site-D could be attributed to the deposition of large SPM containing low estrogen concentrations.

The order of estrogen concentrations in SPM differed from that in filtrated water samples. In the water samples, E1 concentrations were significantly higher than those of E2 and E3, whereas in SPM samples, E1 and E3 concentrations were higher than E2 concentrations. E2, originally excreted by animals, is metabolized to E3 and eventually to E1 [53]. This metabolic pathway typically results in higher E1 concentrations in river water [35,49]. However, in the present study, similar concentrations of E1 and E3 in SPM suggest that E3 had not yet fully metabolized to E1. Therefore, the high E3 concentration in SPM indicates that E3 was initially discharged from animal waste and wastewater.

Huang et al. [31] reported mean estrogen concentrations in SPM of 4.56, 7.95, and 16.5 μg/kg for E1, E2, and E3, respectively, which were higher and followed a different order than those in the present study. In the Huangpu River, China, average concentrations were 81.2, 44.9, and 72.9 μg/kg for E1, E2, and E3 in SPM, respectively, in river water affected by animal feeding operation wastewater [29]. In their study, average estrogen concentrations were less than 20 μg/kg for E1, E2, and E3 in mainstream and tributary mouth waters. Studies by Huang et al. [31] and Nie et al. [29] demonstrated that SPM had high estrogen concentrations primarily due to source factors. In Nie et al. [29], E1 and E3 concentrations were higher than E2, similarly to the present study, whereas in Huang et al. [31], E3 concentrations were higher than both E1 and E2. Sources, SPM characteristics, hydrolysis, and oxidation mechanisms may affect estrogen concentrations in SPM [29,31,53,54,55,56].

Previous studies typically analyzed estrogen concentrations in SPM residues on filter membranes [29,31,34,55], involving small amounts of SPM mass. In the present study, estrogen concentrations were analyzed using a consistent SPM mass of two grams dry weight per analysis. This study collected SPM samples from large water volumes (1000–1500 L) using a high-speed (20,000 rpm) continuous-flow centrifuge. The SPM collection period lasted more than one day, during which estrogen metabolism and decomposition might have occurred, resulting in low estrogen concentrations in SPM. However, analyzing large SPM masses enhances the precision of measurements for very low concentrations of organic compounds such as estrogens in SPM. For instance, Gong et al. [34] used a continuous-flow centrifuge to collect SPM from 250 to 400 L of water. They detected E1 in two of eight SPM samples, with concentrations of 4.7 and 6.0 μg/kg, whereas E2 and E3 were below the detection limit in all samples.

Estrogen concentrations in SPM were converted to the liquid phase. Since colloidal- and soluble-phase estrogen mass fractions were discussed in Section 3.2, this section investigates mass fractions in SPM and liquid phases. In SPM, the average estrogen fractions were 0.16%, 0.21%, and 1.36% for E1, E2, and E3, respectively, suggesting that most estrogens were present in the liquid phase. These fractions were lower than those reported by Huang et al. [31] for the Shaying River, China, where estrogen mass fractions in SPM were 0–19.8%, 0–18.9%, and 0–6.88% for E1, E2, and E3, respectively. In the present study, the difference between total solid and suspended solid was 487.7 mg/L, implying a large amount of dissolved organic matter in the liquid phase, likely binding a high fraction of estrogens. Additionally, SPM discharged from animal wastewater is characterized by low humification and a low adsorption capacity for estrogens.

### 3.4. Multiphase Partition Coefficient of Estrogens

The multiphase partition coefficient is essential for understanding the fate of estrogens in different phases within river systems [6,27,28,29,31,34,55]. This study investigated the organic carbon-normalized estrogen partition coefficients for SPM-filtrate (*K_OC_*), SPM-soluble (*K_POC_*), and colloid-soluble (*K_COC_*) phases, calculated according to Equations (3)–(5) [30,31,34]. Table 3 provides the partition coefficients for the three estrogens. The log *K_OC_* values ranged from 1.08 to 2.96, 2.43 to 3.05, and 2.80 to 4.07 L/kg-C for E1, E2, and E3, respectively. The log *K_POC_* values ranged from 1.23 to 2.97, 2.26 to 3.09, and 2.85 to 4.19 for E1, E2, and E3, respectively. The log *K_COC_* values ranged from 4.23 to 5.26, 4.17 to 5.59, and 4.08 to 5.45 for E1, E2, and E3, respectively. Table 4 outlines the ranges and average estrogen partition coefficients of log *K_OC_*, log *K_POC_*, and log *K_COC_* in this study and as reported in previous studies. Given the limited research on colloid-soluble partition coefficients, log *K_COC_* values from in situ river water and laboratory batch experiments were adopted for comparison.

The order of total average coefficients for log *K_OC_* was 3.38 ± 0.43 (E3) > 2.74 ± 0.33 (E2) > 2.02 ± 0.58 (E1), and for log *K_POC_* it was 3.40 ± 0.41 (E3) > 2.69 ± 0.45 (E2) > 2.06 ± 0.64 (E1). The partition coefficients for log *K_OC_* and log *K_POC_* for E3 were significantly higher than those for E1 (*p* < 0.001). However, the log *K_OC_* and log *K_POC_* values for individual estrogens were not significantly different (*p* = 0.87–0.91). These values were comparable to the ranges reported in previous studies [29,31]. The octanol–water partition coefficient (log *K_OW_*) typically indicates the capacity of estrogens to bind to organic matter [6,53]. In this study, the log *K_OC_* and log *K_POC_* values were independent of the log *K_OW_* values, which are 3.94, 3.13, and 2.85 for E2, E1, and E3, respectively (Table 4). This suggests that the hydrophobicity of estrogens is not the dominant factor controlling their adsorption onto SPM [31,34].

Conventionally, the partition coefficient is the ratio of estrogen concentrations between SPM and liquid phases [31]. In the present study, E1 had significantly higher concentrations than E2 and E3 in the liquid phase, whereas E1 and E3 had higher concentrations than E2 in SPM. This concentration difference resulted in E3 having the highest log *K_OC_* and log *K_POC_* values, and E1 having the lowest. Therefore, the partition coefficients of organic compounds are influenced by their sources and metabolic conditions in both SPM and liquid phases [29,30].

In this study, the total average log *K_COC_* coefficients were 4.72 ± 0.27, 4.75 ± 0.40, and 4.75 ± 0.30 kg/L-C for E1, E2, and E3, respectively. The log *K_COC_* values were comparable to or higher than those studied in the in situ river water and laboratory batch experiments listed in Table 4 [23,24,30,31,33,48,49,57,58]. However, some studies reported higher log *K_COC_* values in in situ river water [22,27,29]. The high log *K_COC_* values may be due to the characteristics of colloids, which have a strong estrogen sorption ability. Log *K_COC_* values obtained in laboratory batch experiments were generally lower than those in in situ river water because laboratory conditions typically involve shorter durations and higher sorbate concentrations [34,59]. Different experimental conditions lead to variations in the partition coefficients of estrogens bound to colloids. Moreover, the log *K_COC_* values were not significantly different among the three estrogens (*p* = 0.88) and were one to two orders of magnitude higher than the log *K_OC_*, log *K_POC_*, and log *K_OW_* values in this study. This is consistent with findings that the estrogen partition coefficients of log *K_COC_* in in situ river water are much higher than log *K_OC_* and log *K_POC_* [29,31]. The high log *K_COC_* values suggest that colloids play a critical role in influencing estrogen distribution, which could further affect estrogen transport, toxicity, and fate in rivers [22,27,28,29,31]. Colloids have a strong ability to absorb estrogen from the liquid phase due to their high specific surface area, sorption site density, organic carbon content, and strong absorption capacity [29,31].

Highly hydrophobic compounds such as PAHs, PCBs, and pesticides have shown a high correlation between log *K_COC_* and log *K_OW_*, suggesting that hydrophobic interactions are the major contributors to the sorption of these compounds to organic matter [6]. However, estrogens, being moderately hydrophobic compounds, might have different sorption mechanisms, forming estrogen–DOM complexes [6,24,51,52,57]. In this study, log *K_COC_* values were not significantly correlated with the hydrophobicity of estrogens (log *K_OW_*) (r = 0.09). Weak correlations between log *K_COC_* and log *K_OW_* for estrogens have been observed in previous studies, suggesting that partition mechanisms other than nonspecific hydrophobic interactions play a key role in estrogen sorption [24,51,52,57]. Previous studies have found that estrogen log *K_COC_* has a strong correlation with the aromaticity and phenolic functional groups of DOM [51,52,57], suggesting that π-π electron interactions, hydrogen bonding, phenolic groups, and ligand exchange play important roles in the binding of estrogens to colloids [6,51,52,59].

### 3.5. Risk Assessment of Estrogens in Filtrate Water and SPM

The ∑EEQ values in the filtrate of water and SPMs are summarized in Table 5. In total, the ∑EEQ values in the filtrate ranged from 3.6 to 142.1 ng/L, with an average value of 43.7 ng/L. In the SPMs, the ∑EEQ values ranged from 0.0 to 40.6 ng/L, with an average value of 6.8 ng/L. The average ∑EEQ values of filtrate were 13.5 and 63.8 ng/L for the years 2013 and 2014 and were 28.5 and 52.3 ng/L for site-U and site-D, respectively. The average ∑EEQ values of SPM were 3.7 and 8.9 ng/L for the years 2013 and 2014, respectively, and were 2.4 and 12.2 ng/L for site-U and site-D, respectively. The ∑EEQ value in site-D was significantly higher than site-U and was significantly different for the years 2013 and 2014, indicating variations in temporal and spatial estrogen risk in river water receiving animal feedlot wastewater. In total (filtrate and SPM), the ∑EEQ values ranged from 4.0 to 182.7 ng/L with an average of 50.5 ng/L. The contribution of ∑EEQ values was 87.4%, and 12.6% was attributed to filtrate water and SPM, respectively.

The estrogenic risk level of ∑EEQ values to organisms is categorized as follows: ∑EEQ < 1 ng/L is a low estrogenic risk, while 1 < ∑EEQ < 10 attributes moderate risk and ∑EEQ > 10 attributes high estrogenic risk. In the present study, the ∑EEQ values of filtrate water were 16% and 84%, respectively, which represent median and high risk for aquatic organisms. The ∑EEQ values of the SPM samples were 40%, 44%, and 16%, which are attributed to low, medium, and high estrogenic risk to aquatic organisms. Furthermore, in water, the contributions of ∑EEQ values were 81.7%, 18.2%, and 0.1% by E1, E2, and E3, respectively. In SPM, the contributions of ∑EEQ values were 78.4%, 11.0%, and 10.6% by E1, E2, and E3, respectively. E1 was the major contributor to the ∑EEQs in the surface water and SPM.

## 4. Conclusions

This study provides important insights into how estrogens behave in river systems affected by animal wastewater. Our findings show that estrogen concentrations vary significantly. E1 is the most dominant in the liquid portion, and higher concentrations are found downstream than upstream. Most estrogens (98.64–99.84%) were discovered in the liquid phase, mainly in the soluble fraction (85.8–87.3%). Our results confirm the original hypothesis that colloids play a crucial role in the distribution of estrogens. The considerably higher log *K_COC_* values than log K_OC_ and log *K_POC_* emphasize the importance of colloidal interactions in influencing estrogen behavior in water environments. We found weak correlations between partition coefficients and log K_OW_, indicating that factors beyond hydrophobicity impact estrogen sorption onto suspended particulate matter and colloids. The risk assessment of E2 estrogen equivalent (EEQ) values suggests that estrogen concentration in water poses a higher risk compared to SPM, with a majority of the samples indicating a high risk to aquatic organisms. The significant role of colloids in estrogen transport suggests that traditional water quality models may underestimate the mobility of these compounds. This study emphasizes the complex interactions between estrogens and different water phases, highlighting the often overlooked role of colloids. It is important to note that our study has limitations, including potential seasonal variations not covered in our sampling period. Further research should investigate temporal dynamics and encompass various aquatic environments to enhance the generalizability of our findings.

## Figures and Tables

**Table 1:** Concentrations of estrogens and DOC of filtrate (<0.45 μm), colloidal (1 kDa-0.45 μm), and soluble (<1 kDa) phases.

Site	Phase	E1, ng/L	E2, ng/L	E3, ng/L	DOC, mg/L
S-U_13_	Filtrate	22.8 ± 11.9	4.1 ± 2.6	5.0 ± 1.6	27.8 ± 9.4
	Colloid	25.6 ± 14.2	5.8 ± 4.8	6.8 ± 3.6	49.2 ± 15.2
	Soluble	16.4 ± 6.7	5.3 ± 4.4	3.8 ± 1.9	20.5 ± 9.7
S-D_13_	Filtrate	46.8 ± 32.5	4.2 ± 3.8	4.0 ± 3.8	11.6 ± 8.6
	Colloid	53.0 ± 47.6	7.9 ± 4.4	6.0 ± 4.6	31.4 ± 13.6
	Soluble	36.7 ± 26.0	5.9 ± 2.7	4.4 ± 3.0	9.0 ± 9.1
S-U_14_	Filtrate	164.7 ± 108.6	6.09 ± 4.50	3.53 ± 1.01	12.4 ± 4.9
	Colloid	239 ± 103	7.8 ± 2.5	5.5 ± 4.3	28.9 ± 19.9
	Soluble	201 ± 93	7.0 ± 5.6	4.0 ± 2.3	12.8 ± 5.0
S-D_14_	Filtrate	328.3 ± 171.0	6.48 ± 2.77	4.63 ± 1.05	4.84 ± 0.6
	Colloid	377.1 ± 219.1	7.9 ± 3.6	6.5 ± 1.6	18.5 ± 3.9
	Soluble	275.3 ± 120.5	4.4 ± 1.4	5.0 ± 0.4	3.0 ± 0.4

S-U_13_, S-U_14_: site-U sampling in 2013 and 2014, respectively; S-D_13,_ S-D_14_: site-D sampling in 2013 and 2014, respectively.

**Table 2:** The SPM concentrations of water samples and TOC and estrogen concentrations in SPM samples.

Site	SPM (mg/L)	TOC (%)	E1 (μg/kg)	E2 (μg/kg)	E3 (μg/kg)
S-U_13_	98 ± 14	33.5 ± 0.1	1.05 ± 1.21	0.30 ± 0.35	0.70 ± 0.81
S-D_13_	35 ± 18	22.3 ± 11.3	2.27 ± 1.34	1.27 ± 1.04	2.13 ± 1.80
S-U_14_	140 ± 32	16.4 ± 1.2	0.72 ± 1.11	ND	0.64 ± 1.28
S-D_14_	40 ± 22	23.1 ± 5.9	1.65 ± 0.44	ND	1.40 ± 0.86

ND: not detected.

**Table 3:** The organic carbon-normalized partition coefficients of log *K_OC_* (SPM-filtrate), log *K_POC_* (SPM-soluble), and log *K_COC_* (colloid-soluble) for estrogens.

Site	Coefficients	E1	E2	E3
S-U_13_	log *K_OC_*	2.41 ± 0.02	2.58 ± 0.03	2.95 ± 0.03
	log *K_POC_*	2.58 ± 0.02	2.42 ± 0.05	3.09 ± 0.11
	log *K_COC_*	4.48 ± 0.19	4.36 ± 0.19	4.57 ± 0.14
S-D_13_	log *K_OC_*	2.54 ± 0.57	3.46 ± 0.05	4.00 ± 0.07
	log *K_POC_*	2.64 ± 0.58	3.52 ± 0.01	4.02 ± 0.11
	log *K_COC_*	4.70 ± 0.13	4.68 ± 0.08	4.70 ± 0.14
S-U_14_	log *K_OC_*	2.30 ± 0.12	NA	3.60 ± 0.07
	log *K_POC_*	NA	NA	3.53 ± 0.11
	log *K_COC_*	4.75 ± 0.41	4.81 ± 0.54	4.94 ± 0.47
S-D_14_	log *K_OC_*	1.39 ± 0.31	NA	3.25 ± 0.21
	log *K_POC_*	1.46 ± 0.29	NA	3.18 ± 0.23
	log *K_COC_*	4.85 ± 0.19	4.99 ± 0.29	4.85 ± 0.17

NA: not available.

**Table 4:** The average and ranges of estrogen partition coefficients of log *K_OC_*, log *K_POC_*, and log *K_COC_* in this study and reported in previous studies.

	E1	E2	E3	Matrix and Method	Ref.
log *K_OW_*	3.43	3.94	2.81		[50]
log *K_OC_*	1.08–2.96 (2.02, 17) *	2.43–3.05 (2.74, 4) *	2.80–4.07 (3.38, 11) *	In situ river water (CFUF)	PS
log *K_OC_*	3.21	3.38	2.84	In situ river water (CFUF)	[31]
log *K_OC_*	1.67–3.46	2.27–2.46	3.53–4.07	In situ river water (CFUF)	[29]
log *K_POC_*	1.23–2.97 (2.06, 14) *	2.26–3.09 (2.69, 4) *	2.85–4.19 (3.40, 13) *	In situ river water (CFUF)	PS
log *K_POC_*	1.75–3.60	2.48–2.60	3.79–4.49	In situ river water (CFUF)	[29]
log *K_POC_*	3.35	3.45	2.94	In situ river water (CFUF)	[31]
log *K_COC_*	4.23–5.26 (4.72, 22) *	4.17–5.59 (4.75, 20) *	4.08–5.45 (4.77, 20) *	In situ river water (CFUF)	PS
log *K_COC_*	4.18–4.85	3.96–4.2		In situ river water (CFUF)	[24]
log *K_COC_*	4.18–4.23	3.96–4.20		River water (CFUF)	[23]
log *K_COC_*	6.60–6.81	6.42–6.78	7.09–7.85	In situ river water (CFUF)	[22]
log *K_COC_*	5.03	4.76	4.25	In situ river water (CFUF)	[31]
log *K_COC_*	5.4		6.11	In situ river water (CFUF)	[22]
log *K_COC_*	7.09	7.58	7.8	In situ river water (CFUF)	[27]
log *K_COC_*	4.08	4.04	4.11	In situ river water (CFUF)	[30]
log *K_COC_*		4.57–4.94		Commercial DOM (FQ)	[48]
log *K_COC_*		4.08–4.68		Biological wastewater (FQ)	[54]
log *K_COC_*		<3–5.25		Biological wastewater (FQ)	[49]
log *K_COC_*	3.98	3.93–4.12		Commercial DOM (FQ)	[55]
log *K_COC_*	4.52–6.02	3.42–5.11		Commercial DOM (SPME)	[33]

PS: present study, FQ: fluorescence quenching; CFUF: cross-flow ultrafiltration; SPME: solid-phase microextraction. * (mean value, sample numbers).

**Table 5:** EEQ values (ng/L) of the filtrate and SPM in Wulo Creek.

Species	EEQ of Filtrate		EEQ of SPM	
	Mean ± SD	Ranges	Mean ± SD	Ranges
E1	38.2 ± 38.4	3.1–132.3	9.1 ± 12.4	0.4–40.6
E2	5.4 ± 3.6	0.0–16.7	3.6 ± 1.5	2.2–5.1
E3	0.0 ± 0.0	0.0–0.1	0.0 ± 0.0	0.0–0.0
Sum EEQ	43.7 ± 40.6	3.6–142.1	6.8 ± 11.0	0.0–40.6

## Data Availability

Data are available through request to the corresponding author.

## Supplementary Materials

The following supporting information can be downloaded at https://www.mdpi.com/article/10.3390/toxics12090671/s1: Table S1: Basic water chemistry of Wulo Creek. Table S2: Quality assurance data for the analysis of each target compound in DI water and river water.
