# Supplementary material for: Multiphase Partitioning of Estrogens in a River Impacted by Feedlot Wastewater Discharge
Source: Toxics. 2024 Sep 14;12(9):671. doi: 10.3390/toxics12090671 (PMC11436132; doi:10.3390/toxics12090671)
Supplement: Supplementary file 1 [file toxics-12-00671-s001.zip › toxics-3137327-supplementary.pdf]

Supplementary information:

Sampling strategy

The sampling was conducted over a period of two years to ensure robust data collection during the dry season from January to April. Each month, two samples were collected from both upstream and downstream locations, resulting in a total of 16 samples. However, due to sampling failures, such as technical issues with the centrifugation process for SPM and liquid phase samples (as described in Section 2.3), and challenges in achieving the desired mass balance for colloid and dissolved phase separation of DOC and estrogen (exceeding 100±60%), only 10 valid samples were obtained in the year 2013, with 4 from upstream and 6 from downstream.

As a result of these challenges, it was decided to continue the sampling in the year 2014 using the same procedures as the previous year, with two samples collected from each location every month, resulting in a total of 15 valid samples (9 from upstream and 6 from downstream). In total, there were 25 valid samples, including 13 from upstream and 12 from downstream.

Table S1. Basic water chemistry of Wulo Creek

		pH	SS, mg/L	COD, mg/L	NH_3-N, mg/L	T, ^{0}C	EC, μS/cm
Total	min	6.84	17.3	6.0	4.33	21.0	641
	max	9.16	187.8	187.0	24.60	31.1	851
	mean	7.48	77.8	71.1	9.57	26.1	715
	SD	0.67	51.7	46.2	4.36	2.2	78
Site U	mean	7.63	118.5	108.4	11.51	25.5	750
	SD	0.67	40.5	33.2	5.28	2.2	99
Site D	mean	7.34	37.0	61.5	7.63	26.6	681
	SD	0.30	18.6	11.8	1.80	2.1	30
p value		0.11	<0.001	<0.001	0.006	0.12	0.20

Table S2. Quality assurance data for the analysis of each target compound in DI water and river water.

	Recovery (%)		MDL
	River water	DI water	ng/L
E1	79	93	0.3
E1-d4	80	94	0.3
E2	89	96	0.5
E2-d5	87	96	0.5
E3	91	99	0.4

From: Occurrence and degradation of free and conjugated estrogens in a river receiving feedlot animal discharge (Hung et al.[38])
